# Nursing Home Social Workers Perceptions of Preparedness and Coping for COVID-19

**DOI:** 10.1093/geroni/igaa057.3487

**Published:** 2020-12-16

**Authors:** Vivian Miller, Noelle Fields, Nancy Kusmaul, Keith Anderson, Christy Maxwell

**Affiliations:** 1 Bowling Green State University, Bowling Green, Ohio, United States; 2 The University of Texas at Arlington, Arlington, Texas, United States; 3 UMBC, Baltimore, Maryland, United States; 4 University of Texas at Arlington, Arlington, Texas, United States

## Abstract

Social work has a long history of responding to the needs of vulnerable populations during times of crisis and disaster. Social workers are working at the front lines responding to the current COVID-19 pandemic in a variety of health care practice settings, including nursing homes, however it is unclear how social workers perceive their preparedness during this time. This study employed a cross-sectional survey to nursing home social workers via social media on feelings of preparedness for COVID-19, what has been most professionally helpful for social workers during these times in their role in COVID-19, as well as demographic questions. Demographic data were analyzed using SPSS and qualitative data were analyzed using the rigorous and accelerated data reduction (RADaR) technique. Data are based on a sample of 63 (N=63) nursing home social workers. Findings revealed that while some social workers felt prepared for the coronavirus, many respondents stated that they were unprepared to meet the demands and challenges they were facing. Moreover, participants shared that professional support was critically important to get through COVID-19. These findings are important, as social workers are tasked with ensuring each resident attains their highest level of psychosocial well-being, which can be achieved only when nursing home staff are supported. Findings from the present study suggest that additional support for nursing home staff ought to include peer mentoring and mutual support. Additionally, improved leadership across health care settings is worth assessing.

